# Supplementary material for: ChatGPT as a Tool for Biostatisticians: A Tutorial on Applications, Opportunities, and Limitations
Source: Stat Med. 2025 Oct 23;44(23-24):e70263. doi: 10.1002/sim.70263 (PMC12548020; doi:10.1002/sim.70263)
Supplement: Supplementary file 1 — Data S1. Supporting Information. [file SIM-44-0-s001.zip › Supplementary Materials Additional.zip!/3.7_Translation_Between_Multiple_Statistical_Programming_Languages/3.7.2_SPSS_to_R/3.7.2_Translation_Chat5.pdf]

R 5

Clear memory

I don't have memory on in this chat, so I can't store any information you've shared in past conversations. If you'd like to manage or clear memory in the future, you can do that by going to **Settings > Personalization > Memory**.

Let me know if you want help navigating there or if there's something else on your mind!

Take the role of an expert biostatistician for this chat

Got it — stepping into the role of an expert biostatistician. Whether you're working on clinical trials, epidemiological studies, or bioinformatics data, I've got you covered. Just let me know what you're working on or what you need help with — design, analysis, interpretation, statistical modeling, you name it.

I am running the following analysis in SPSS:
DATASET ACTIVATE DataSet5. LOGISTIC REGRESSION VARIABLES V2 /METHOD=FSTEP(LR) V3 V4 V5 V6 V7 V8 V9 V10 V11 V12 V13 V14 V15 V16 V17 V18 V19 V20 V21 V22 V23 V24 V25 V26 V27 V28 V29 V30 V31 V32 /CRITERIA=PIN(0.05) POUT(0.10) ITERATE(20) CUT(0.5).
Translate this into R code.
The data is loaded into R with
temp <- tempfile()
download.file(
 "https://archive.ics.uci.edu/static/public/17/breast+cancer+wisconsin+diagnostic.zip",
 temp
)
data <- read.table(unz(temp, "wdbc.data"), sep = ",")
data$V2 <- data$V2 == "M"
unlink(temp

Great — you're running a **stepwise logistic regression** in SPSS using **forward selection (likelihood ratio)** and a p-value threshold for entry/removal (PIN = 0.05, POUT = 0.10), with your dependent variable being `V2`, and a set of predictors from `V3` to `V32`.

Let's translate this to R using `glm()` for the logistic model and `step()` for stepwise selection.

Here's how to do that in R:

```r
# Recode V2 as binary (already done in your script)
data$V2 <- data$V2 == "M"  # TRUE for malignant, FALSE for benign

# Fit null model (intercept only)
null_model <- glm(V2 ~ 1, data = data, family = binomial)

# Fit full model with predictors V3 to V32
full_model <- glm(V2 ~ ., data = data[, c("V2", paste0("V", 3:32))], family = binomial)

# Stepwise selection: forward (based on AIC by default; we'll mimic p-value criteria with direction
# Note: R's step() uses AIC, but to mimic PIN/POUT thresholds you would need a custom approach
# For now, we'll do standard forward selection using step()
step_model <- step(null_model,
                   scope = list(lower = null_model, upper = full_model),
                   direction = "forward",
                   trace = TRUE)

# Summary of the final model
summary(step_model)
```

Notes:

- **SPSS's FSTEP(LR)** uses likelihood ratio tests for entry/removal, and stepwise selection based on p-values. R's `step()` uses **AIC** by default, which may not yield exactly the same model.
- To more closely match **PIN/POUT**, you could write a custom selection function based on likelihood ratio tests and p-values, but for many practical purposes, AIC-based stepwise selection gives a very similar model.

Would you like me to write a custom p-value-based stepwise selection function to match SPSS behavior more closely?

Yes

Perfect. Let's build a **custom forward stepwise logistic regression** in R based on **likelihood ratio tests**, mimicking SPSS's `FSTEP(LR)` behavior with:

- **PIN (p-inclusion)** = 0.05
- **POUT (p-removal)** = 0.10
- We'll only implement **forward selection**, as you did in SPSS.

Here's the R code for that:

```r
# Ensure V2 is binary
data$V2 <- data$V2 == "M"

# Define response and predictor variables
response <- "V2"
predictors <- paste0("V", 3:32)
included <- c()  # Variables currently in the model
remaining <- predictors  # Variables not yet in the model
```

```r
# Set PIN and max iterations
pin <- 0.05
max_iter <- 20
iter <- 0
improved <- TRUE

while (improved && iter < max_iter) {
  iter <- iter + 1
  p_values <- c()

  # Test adding each remaining variable one at a time
  for (var in remaining) {
    current_formula <- as.formula(
      paste(response, "~", paste(c(included, var), collapse = " + "))
    )
    base_formula <- if (length(included) == 0) {
      as.formula(paste(response, "~ 1"))
    } else {
      as.formula(paste(response, "~", paste(included, collapse = " + ")))
    }

    full_model <- glm(current_formula, data = data, family = binomial)
    base_model <- glm(base_formula, data = data, family = binomial)

    # Likelihood ratio test
    lr_test <- anova(base_model, full_model, test = "LRT")
    p_val <- lr_test$`Pr(>Chi)`[2]
    p_values <- c(p_values, p_val)
  }

  names(p_values) <- remaining

  # Find the best candidate with lowest p-value
  min_p <- min(p_values, na.rm = TRUE)
  best_var <- names(which.min(p_values))

  if (!is.na(min_p) && min_p < pin) {
    included <- c(included, best_var)
    remaining <- setdiff(remaining, best_var)
    cat(sprintf("Step %d: Added %s (p = %.4f)\n", iter, best_var, min_p))
  } else {
    improved <- FALSE
  }
}

# Final model
final_formula <- as.formula(paste(response, "~", paste(included, collapse = " + ")))
final_model <- glm(final_formula, data = data, family = binomial)

# Summary of the selected model
summary(final_model)
```

What this script does:

- Starts with an intercept-only model
- Iteratively evaluates all unused predictors using likelihood ratio tests
- Adds the variable with the lowest p-value if it's below 0.05 (your `PIN`)
- Stops when no variables meet the criteria or the iteration limit is hit

Let me know if you'd like the reverse (backward) or full stepwise version too — or if you want AUC/cross-validation added for validation.
